# Anterior cingulate cortex involved in social food‐foraging decision‐making strategies of rats

**DOI:** 10.1002/brb3.768

**Published:** 2017-09-08

**Authors:** Xiaolin Zhong, Sihao Deng, Wenbo Ma, Yuchen Yang, Dahua Lu, Na Cheng, Dan Chen, Hui Wang, Jianyi Zhang, Fang Li, Changqi Li, Hua‐Lin Huang, Zhiyuan Li

**Affiliations:** ^1^ Key Laboratory of Regenerative Biology Guangdong Provincial Key Laboratory of Stem Cell and Regenerative Medicine Guangzhou Institute of Biomedicine and Health Chinese Academy of Sciences Guangzhou China; ^2^ Department of Anatomy and Neurobiology Xiangya School of Medicine Central South University Changsha Hunan China; ^3^ GZMU‐GIBH Joint School of Life Sciences Guangzhou Medical University Guangzhou China

**Keywords:** anterior cingulate cortex, food‐foraging, neural processing, social decision‐making

## Abstract

**Introduction:**

Decision making as a complex cognitive process involves assessing risk, reward, and costs. Typically, it has been studied in nonsocial contexts. We have developed a novel laboratory model used with rodents to detect food‐foraging decision‐making strategies in different social settings. However, the brain regions that mediate these behaviors are not well identified. Substantial evidence shows that the anterior cingulate cortex (ACC) participates in evaluation of social information and in decision making.

**Methods:**

In this study, we investigated the effect of bilateral lesions in the ACC on established behaviors. Kainic acid (KA) was administered bilaterally to induce ACC lesions, and saline microinjection into the ACC was used in the sham group.

**Results:**

In contrast to the sham‐lesioned animals, when faced with the choice of foraging under a social context, rats with ACC lesions preferred foraging for the less desirable food. Moreover, in these situations, the total amount of food foraged by the ACC‐lesioned group was less than the amount foraged by the sham group. Notably, neither social interactions nor social agonistic behaviors were affected by ACC lesions.

**Conclusions:**

These data suggest that the ACC is a key region underlying neural processing of social decision‐making, specifically tending to compete for foraging high predictive reward food.

## 1 INTRODUCTION

Decision‐making can be defined as the process of selecting an option from several schemes, and the option that the decision maker chooses must lead to the maximal benefit for the individuals (Floresco, St Onge, Ghods‐Sharifi, & Winstanley, 2008). For social animals, including humans, purely individual decision‐making is rare; most decisions are made in social settings (Seo & Lee, 2012), which include other individuals and groups. A growing body of human studies investigated the effect of social context on decision‐making (Fatfouta, Schulreich, Meshi, & Heekeren, 2015; FeldmanHall, Raio, Kubota, Seiler, & Phelps, 2015; Heijne & Sanfey, 2015). For example, Heijne and Sanfey (2015) reported that the effect of prior beliefs on stay/leave decision‐making was much less pronounced in a social than in a nonsocial context. For rodents, which are highly social experimental animals, social situations affected their decision‐making including mate choice, territorial scramble and food foraging (Dall & Wright, 2009; Robinson, Feinerman, & Franks, 2012; Westneat, Walters, McCarthy, Hatch, & Hein, 2000). The findings from Hillman and Bilkey (2012) suggested that the anterior cingulate cortex (ACC) is an important cost–benefit domain for encoding spatial and competitive decision‐making in rats. For food foraging, we have established a novel paradigm to investigate decision‐making in a model animal, with or without a mimetic desire paradigm; glutamatergic and dopaminergic systems play various roles in these processes (Li et al., 2016). However, the brain regions that mediate these behaviors have not been pinpointed. Is the little neural‐level investigation ACC?

The ACC lies on the medial surface of the frontal lobe and forms one of the largest parts of the limbic system. Previous studies demonstrated that the ACC was associated with motivation (Holroyd & Yeung, 2012) and cost‐benefit decisions (Rudebeck, Walton, Smyth, Bannerman, & Rushworth, 2006; Schweimer & Hauber, 2005; Walton, Bannerman, Alterescu, & Rushworth, 2003; Walton, Bannerman, & Rushworth, 2002) in a nonsocial context (Holroyd & McClure, 2015). To date, the role of the ACC in encoding decision‐making under social food‐foraging contexts has not been well examined. Recent findings suggested that the ACC gyrus was involved in social information valuation (Rilling et al., 2002; Rudebeck, Buckley, Walton, & Rushworth, 2006; Tomlin et al., 2006). Studies have reported that the ACC plays an important role in both social‐based decision‐making (de Araujo et al., 2012; Steinmann et al., 2014) and value‐based decision‐making (Apps & Ramnani, 2014; Massar, Libedinsky, Weiyan, Huettel, & Chee, 2015; Vassena, Krebs, Silvetti, Fias, & Verguts, 2014). We have demonstrated that the ACC mediates food foraging‐related behaviors (Misakian & Kaune, 1990). So, a plausible hypothesis is that the ACC is capable of encoding social‐based and value‐based food foraging decision‐making behaviors.

In the current study, with an irreversible lesion induced by intra‐ACC kainic acid (KA) infusion used previously (Li et al., 2012), which was supported be the elaborated findings from the research of Dall, Westneat, and Hillman et al., we investigated the role of the ACC in food foraging decision‐making behaviors under different social conditions (Li et al., 2016). Our results showed that rats with ACC lesions preferred foraging for the less desirable food when faced with the choice of foraging in a competitive environment, which as sham‐lesioned animals preferred foraging for the more desirable food. We also found that the total amount of food foraged in the ACC‐lesioned group was less than in the sham‐lesioned group. Furthermore, we tested the specificity of the model with parallel social interaction tests and tube tests, and neither social interaction nor social agonistic behaviors were affected by ACC lesions. These findings indicated that the ACC might play a key role in mediating the food foraging decision‐making behaviors under different social contexts, specifically tending to compete for foraging high predictive reward food.

## 2 MATERIALS AND METHODS

### 2.1 Animals

Adult nonfamiliar male Sprague–Dawley rats, weighing 250–300 g, were obtained from the inbred strain animal center (Xiang‐Ya School of Medicine, Central South University, Changsha, China). All rats were housed together (*n* = 3 per 50 cm × 35 cm × 20 cm cage), and they were adapted to their new environment for 1 week after arrival. The animal house was maintained at 23 ± 2°C, 50 ± 5% humidity and 12 hr light/dark. The experimental protocol was approved by the Animal Care and Use Committee of Central South University and conformed to the National Institutes of Health Guide for the Care and Use of Laboratory Animals. All efforts were made to minimize both the number and the suffering of rats used. For each group in this series of experiments, 7–18 animals were used.

### 2.2 Surgical procedure

Surgery was performed using a rat brain stereotactic apparatus (RWD, Shenzhen, China). All rats were anesthetized with an intraperitoneal injection of chloral hydrate at the dose of 40 mg/kg. Kainic acid (KA) monohydrate (Sigma) was dissolved in sterile saline, final concentration 1 mg/ml, and adjusted to pH 7.2–7.4, using 0.1 mol/L NaOH. Microinjection of KA solution was administered into the ACC as described in our previous studies (Li et al., 2012) and other reports (Ren et al., 2008). Briefly, anesthetized rats were fixed on the brain stereotactic apparatus; the skull was exposed, and burr holes were drilled above the ACC. A 28 gauge injection cannula (Plastics One, Roanoke, VA) was introduced stereotactically and directed toward the ACC. Rats received bilateral injections of either saline (*n* = 16) or KA (*n* = 22) at the following coordinates (relative to bregma), AP +2.7 mm, ML ± 0.5 mm, DV ‐2 mm and AP +1.7 mm, ML ± 0.5 mm, DV ‐2.8 mm from the dura. Each infusion delivered 0.3 μl KA or NS (control animals).The microinjection lasted for 5 min; the cannula was left in place for an additional 5 min to allow for complete diffusion. Penicillin powder was used to prevent infection. After surgery, rats were placed in a warm environment until they regained consciousness; then, they were allowed to recover in their home cages for 3 weeks.

### 2.3 Social food foraging decision‐making behaviors

All behavioral tests were carried out 3 weeks after ACC lesion surgery. Social food foraging decision‐making behaviors were tested as described previously (Li et al., 2016) with some modifications that the reward food or the resident rats were added in some cages as an appetitive behavior or a social conflict, respectively. Briefly, 1 day before the test, to eliminate contact between the rats during the food deprivation, rats were housed individually in a small cage (30 cm × 18 cm × 16 cm, length × width × height). The rats were deprived of food for 12 hr; then, they were placed in an open field for the food foraging decision‐making behavior test, which was constructed with black wood with dimensions of 150 cm × 150 cm × 50 cm. Tests began at 17:00 every day; the test rats were removed from their home cages, placed in the open field apparatus and allowed to move freely from 17:00 to 19:00 to habituate to the environment, and then, two small plastic cages with 200 g of food pellets on the metal, removable wire lids were placed into the open field. The test rat was allowed to navigate to the cages and forage food pellets freely from 19:00 to 21:00. The social or nonsocial contexts were designed as follows:

#### 2.3.1 Condition 1

**Figure 1:**
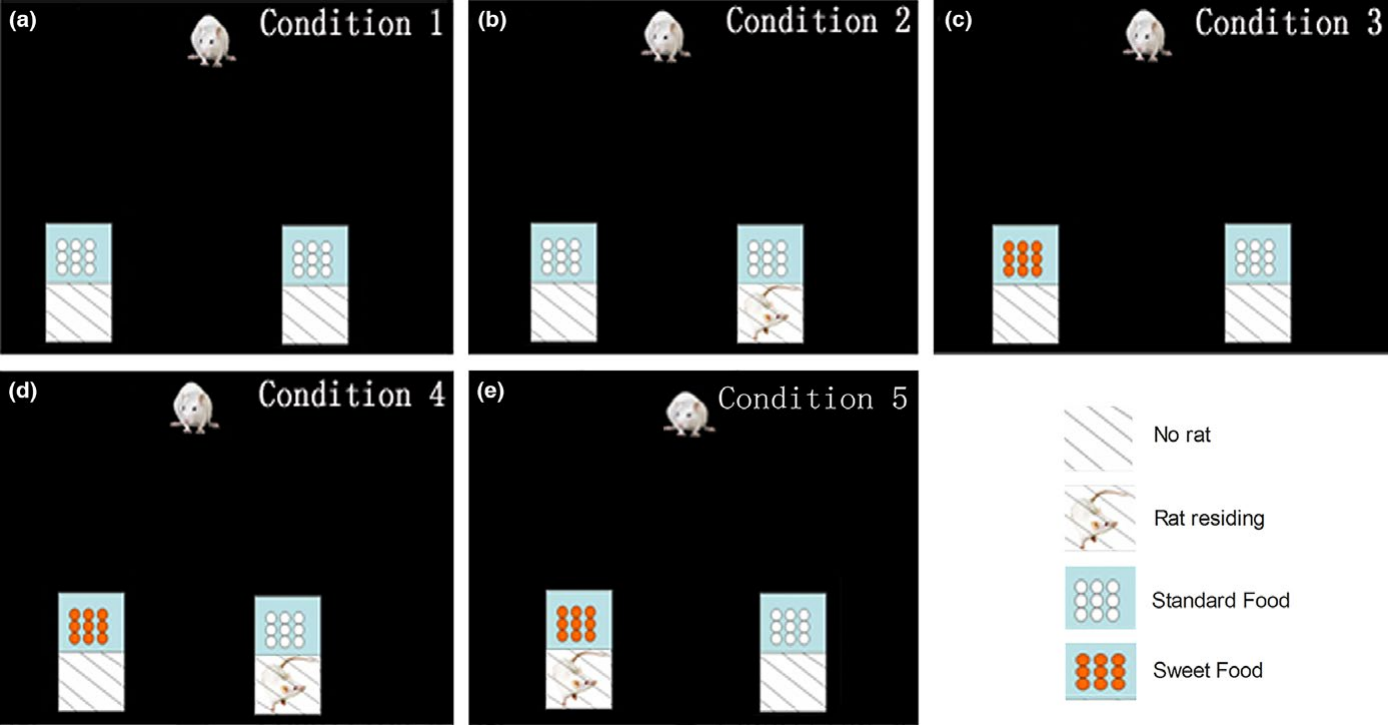
Schematic photographs of the food foraging decision‐making paradigm designs in different contexts. Schematic photographs of the food foraging decision‐making paradigm designs in Condition 1 (a), Condition 2 (b), Condition 3 (c), Condition 4 (d) and Condition 5 (e). Two small wire‐topped plastic home cages (30 cm×18 cm×16 cm) were placed in the open field during tests. The rat in the small cage (in Condition 2, Condition 4 and Condition 5) was allowed to reside in the cage for at least 1 week before the experiment. On each trial, the rat in the open field had to make choices between the two cages from which food could be foraged

The two small cages were both empty, and standard food pellets were placed on the removable wire lids (Figure 1a).

#### 2.3.2 Condition 2

One of the small cages was empty, and the other had a resident rat of the same sex stranger (male) as the test rat, and standard food pellets were on the removable wire lids of both cages. (There were two removable wire lids on the cage that had a resident rat to isolate that rat from the food pellets on the container to calculate the amount of food eaten by the test rats, but the rat was permitted exchange of olfactory, visual, and tactile cues for shaming a food competitor.) (Figure 1b).

#### 2.3.3 Condition 3

Neither small cage had a rat inside, but one cage had standard food pellets and the other had sweet (10% sugar) food pellets on the removable wire lid (Figure 1c).

#### 2.3.4 Condition 4

There were sweet food pellets on the empty cage and standard food pellets on the cage with a same sex stranger rat inside (Figure 1d).

#### 2.3.5 Condition 5

There were standard food pellets on the empty cage and sweet food pellets on the cage with a same sex stranger rat inside (Figure 1e).

After a two‐hour test, the amount of food pellets left in the food containers was calculated separately. The total amount of foraged food was calculated using the formula: 400 g ‐ the amount of food left in both of the food containers. The percentage of foraged food pellets from a single food container was calculated using the formula: (200 g ‐ the amount of food left in this food container)/total amount of foraged food. The amount of food eaten was calculated using the formula: 400 g ‐ the amount of food pellets moved onto the open field ‐ the amount of food left in both of the food containers.

### 2.4 Social interaction test

The social interaction test commenced as reported in our previous studies (Cao et al., 2013) and other published methods (Holroyd & McClure, 2015; Tajerian et al., 2014). The apparatus consisted of a large (120 × 48 × 54 cm^3^) three‐chamber glass testing arena. One large compartment was in the middle, and two small boxes (28 × 28 × 12.5 cm^3^) were located on either side. The large compartment and the small boxes were separated by wire‐mesh barriers, which permitted exchange of olfactory, visual, and tactile cues but prevented aggressive interactions and also prevented the “stranger” rat in the small box from initiating the social contact. The large compartment was divided into three parts. The center part was defined as the nonsocial preference area; the other two parts were preference areas; the side with a same‐sex stranger rat in the small box was called the social target side; and the side without rat in the small box was called the inanimate target side.

The experiment included two phases: the adaptation phase and the social preference test phase. In the adaptation phase, after the test rats habituated in the test room for 30 min, they were placed in the middle of the compartment of the social interaction box and allowed to freely explore the compartment for 8 min daily for 4 days. During this phase, there were no same‐sex stranger rats in any of the small boxes. The amount of time spent in each part was used as baseline data to assess general activity. The social preference test was performed on the fifth day. During this time, a stranger rat was placed in one of the small boxes. Then, the test rats were placed in the middle of the compartment and moved freely to explore the two small boxes for 10 min. At the end of each test, all animals were removed from the apparatus, and the entire testing arena was washed with water and ethanol (75%) to remove residual odors. All tests were conducted daily between 14:30 and 17:30 and recorded by a video camera. The amount of time recorded in the preference area began when all four paws of the experimental animal crossed over the taped boundary line and terminated as soon as one paw crossed over the boundary line back into the central compartment. Preference behaviors were evaluated as the cumulative time spent in the social target side or inanimate side and as the number of contacts with each wire‐mesh. The person who analyzed the time spent and number of contacts was blind to the groups.

### 2.5 Tube test

The tube test assay was adapted from previous studies (Wang et al., 2011) with some modifications. The apparatus was a transparent tube 1.5 m long with a 6 cm inside diameter, a size just sufficient to permit one adult rat to pass through without reversing direction. Before the test, all rats were weighed, and an ACC‐lesioned rat and a weight‐matched (weight difference no greater than 20 g) sham‐lesioned rat were selected as a group. During the test, the two weight‐matched rats were released simultaneously into opposite ends of the tube, and care was taken to ensure that they met in the middle of the tube. The first rat to retreat from the tube within the 2 min test was designated the “loser”. In cases when neither rat retreated within 2 min, the test ended as a draw. Between trials, the tube was cleaned with 75% ethanol. The evaluation index was the percentage of winning, which was calculated by the formula: the number of wins/total number of tests × 100%.

### 2.6 Nissl‐staining

After the behavioral tests, ACC‐lesioned and sham‐lesioned rats were euthanized by intraperitoneal injection of an overdose of 10% chloral hydrate (80 mg/kg) and were then perfused transcardially with 100 ml 0.9% saline followed by 300 ml ice‐cold 4% paraformaldehyde solution (pH = 7.4). Brains were removed and postfixed for 24 hr in 4% paraformaldehyde at 4°C, and then cryoprotected with 15% phosphate‐buffered sucrose overnight; the next day, the brains were cryoprotected with 30% phosphate‐buffered sucrose (pH = 7.4). Coronal brain sections (30 μm) were cut with a cryostat at −24°C, and one set containing thirty slices of ACC was randomly obtained from each rat at +2.70 mm to +0.70 mm rostrocaudal from bregma. The brain sections were processed for Nissl staining. Free‐floating sections were washed with 0.01 mol/L phosphate‐buffered saline (PBS) and mounted on slides. After the slides were degreased and dewaxed, the Nisslstaining solution (Sigma, USA) was added on the sections; 30 min later, the Nissl staining solution was removed. The slides were dehydrated through graded alcohols (70, 80, 90, and 100% × 2) placed in xylene and cover slipped, using Histo Mount mounting medium. This experiment was performed to determine the extent and location of the KA‐induced lesions. Images were subsequently captured using a Nikon light microscope (H600 L Light Microscope, Japan). The person who analyzed the histology was blind to the treatment.

### 2.7 Statistical analysis

Statistical analyses were performed using Graph Pad Prism 5.01 software (GraphPad Software, San Diego, CA, USA). The results are presented as the mean ± SEM. Significant differences were determined using either Student's *t* test for two‐group comparisons or ANOVA followed by post hoc Dunnett testing for multiple comparisons between more than two groups. Differences between groups were compared using two‐way analysis of variance (ANOVA) followed by Bonferroni testing where appropriate. For the tube test, a chi‐square was used to compare the percentage of winning. A value of *p* < .05 was considered statistically significant.

## 3 RESULTS

### 3.1 Examination of KA‐induced lesions in the ACC using Nissl‐staining

**Figure 2:**
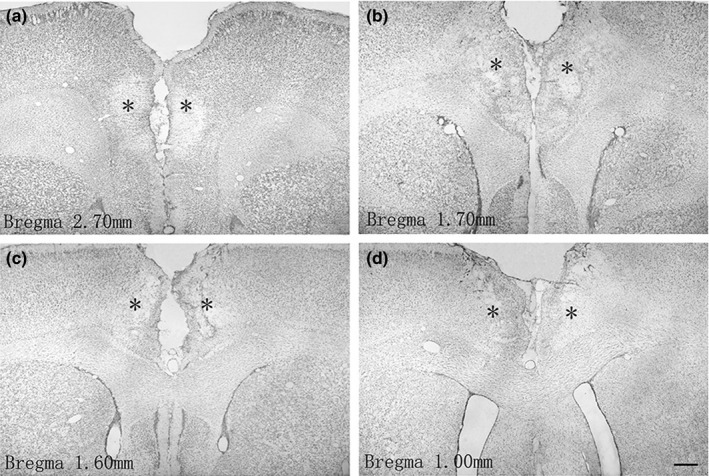
Representative photographs of the extent of the anterior cingulate cortex (ACC) lesions in selected rats. Representative photographs of coronal sections of rat brain show the extent of bilateral ACC lesions induced by KA injection at 2.70 mm (a), 1.70 mm (b), 1.60 mm (c) and 1.00 mm (d) anterior to the bregma, respectively. * shows the area of induced ACC lesion

Nissl‐staining was used to examine the lesions of the ACC after bilateral microinjections of KA, which could lead to cell loss throughout the entire area. In contrast to four excluded lesioned rats for their too small lesions, five sham rats (microinjection of NS into the ACC) were excluded from the statistical analysis because their obvious damage, caused by the injection cannulae during vehicle microinfusion. Finally, 16 rats in the sham group displayed minimal damage (data not shown), whereas KA‐treated rats (*n* = 22) showed an obvious neural lesion at and around the injection site in the ACC (Figure 2). The ACC lesions were targeted and reproducible in all cases.

### 3.2 ACC lesions‐induced selective alterations in food‐foraging decision‐making behaviors in rats

**Figure 3:**
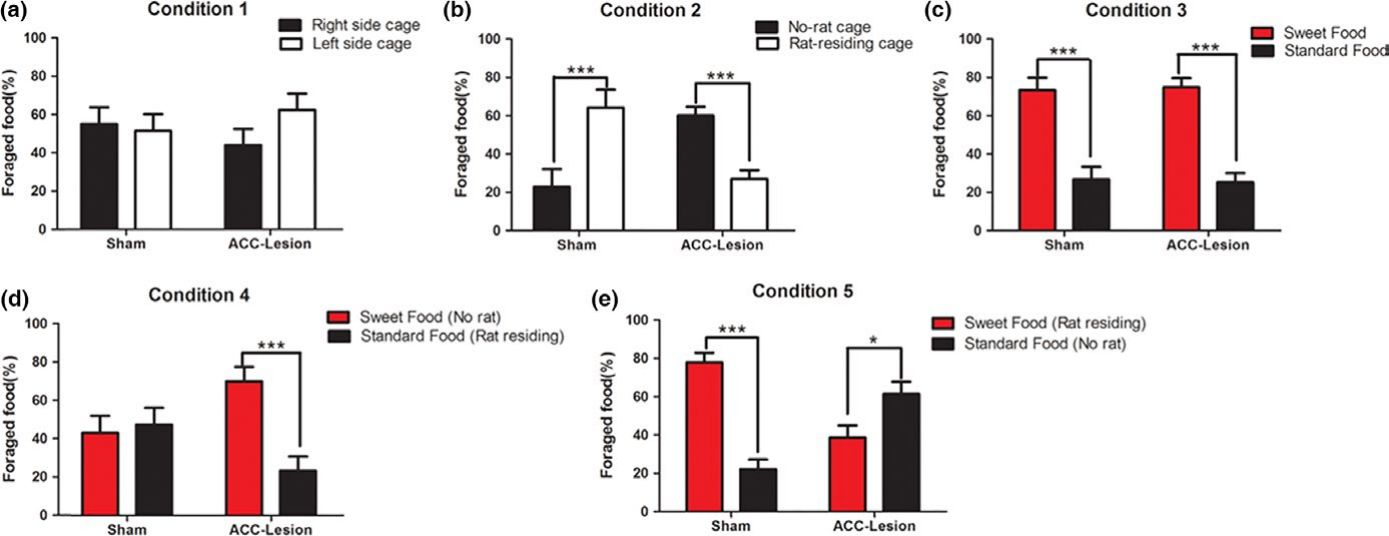
Effect of anterior cingulate cortex (ACC) lesions on the food foraging decision‐making behaviors with or without social information. The percentage of food foraged from the two small cages in Condition 1 (a), Condition 2 (b), Condition 3 (c), Condition 4 (d) and Condition 5(e). The percentage of foraged food from either side cage in Condition 1 was not significantly different for both the sham and the ACC‐lesioned animals. In Condition 2, for sham‐lesioned rats, the percentage of foraged standard food pellets from the resident rat cage dramatically increased relative to the empty cage; whereas for ACC‐lesioned animals, the percentage of food foraged from the cage with a resident rat was less than from the empty cage. Both the sham and the ACC‐lesioned groups preferred foraging for sweet food pellets in Condition 3. The sham group showed no significant difference between the percentage of standard food pellets foraged from the resident rat cage and sweet food pellets foraged from the no‐rat cage, whereas for the ACC‐lesioned rats, the percentage of sweet food foraged from the empty cage was higher than the percentage of standard food pellets from the resident rat cage in condition 4. In Condition 5, the percentage of the sweet food that the sham‐lesioned animals foraged from the cage with a rat resident was higher than the standard food from the empty cage, but there was no significant difference between the percentage of sweet food pellets foraged from the cage with a rat resident and standard food pellets foraged from the empty cage for the ACC lesion group. Here, *n* = 13 for the sham group, and *n* = 18 for the ACC‐lesioned group. ***p < .001; *p < .05

We challenged rats to make decisions in five different contexts as they performed food foraging tasks in which they could turn right or left to forage for food. The tasks presented the animals with different combinations of social conflict and value benefits, with standard food or sweet food on the cage as benefits and a same‐sex stranger rat in a cage as a conflict that the ‘residing rat’ were used for shaming a food competitive environment (Figure 3).The aim of this experiment was to determine whether ACC lesions alter the decision‐making in the five different conditions we designed.

Condition 1 was a control situation. Two‐way ANOVA indicated there were no main effects of ACC lesions (*F* (1, 32) = 0.0000, *p > *.05), the side of cage (*F* (1, 32) = 0.7454, *p > *.05), or their interaction (*F* (1, 32) = 1.624, *p > *.05) (Figure 3a) on the percentage of food foraged from the cages on the left or the right sides by both groups of animals. However, in Condition 2, there was a significant interaction between the side of cage and the ACC lesions (*F* (1, 32) = 1.624, *p < *.05) (Figure 3b) affecting the percentage of food foraged in this social environment. Bonferroni posttests showed that the sham‐lesioned rats foraged a higher percentage of food from the cage with a resident rat than from the cage with no rat (*p < *.001) (Figure 3b). In contrast, the ACC‐lesioned group foraged a lower percentage of food from the cage with a resident rat than from the empty cage (*p < *.001) (Figure 3b). These results indicated that, in Condition 2, when ACC‐lesioned rats were faced with the choice of foraging in a competitive environment, they preferred foraging for the food the sham‐lesioned rats found less desirable. In Condition 3, we used sweet food (containing 10% sugar) as well as standard food. Using two‐way ANOVA, we found a significant main effect for food type (standard or sweet) (*F* (1, 36) = 71.97, *p < *.0001) (Figure 3c) on the percentage of food foraged from cages in both the ACC‐lesioned group and sham‐lesioned group, suggesting that ACC lesions did not affect the rat's preference for sweet food.

**Figure 4:**
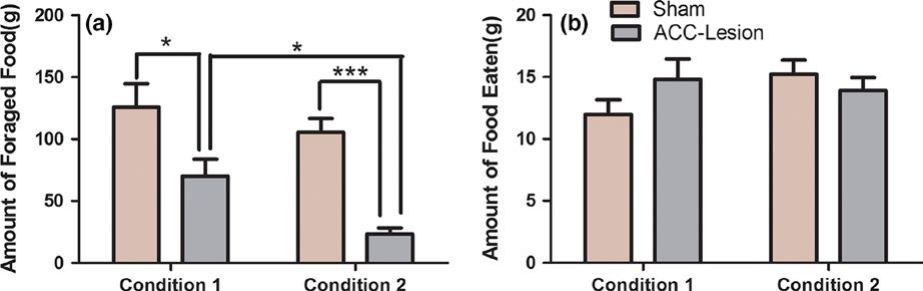
Effect of anterior cingulate cortex (ACC) lesions on the amount of food foraged and eaten under nonsocial or social conditions. (a) The amount of food foraged by the ACC‐lesioned and the sham groups in Condition 1 and Condition 2. (b) The amount of food eaten in the ACC‐lesioned and sham groups in Condition 1 and Condition 2. Both ACC lesions and social environment affected the amount of food foraged. However, the amount of food eaten was not affected by ACC lesions or social environment. ***p < .001; *p < .05

In Condition 4, Bonferroni posttests showed that there was no significant difference between sweet and standard food pellets in the percentage of food foraged by the sham group (*p > *.05, *n* = 8), whereas in the ACC‐lesioned group, more sweet food was foraged from the empty cage than standard food from the cage with a resident rat (*p < *.0001, *n* = 9) (Figure 3d).In contrast, in Condition 5, the sham‐lesioned rats preferred to forage sweet food from the cage with a resident rat than standard food from the empty cage (*p* < .0001, *n* = 12); however, the ACC‐lesioned rats foraged a higher percentage of food from the empty cage with the standard food than from the cage that had a resident rat and sweet food (*p* < .05, *n* = 12) (Figure 3e). Finally, in combinatorial Conditions 4 and 5, the two‐way ANOVA indicated that the percentage of foraged food was affected by food type was less than the interaction between food type and the ACC lesions (*F* (1, 38) = 7.264, *p* < .05), but as well as the interaction between the food type and the normal ACC function (*F* (1, 38) = 0.06946, *p* > .05).To further determine the role of ACC lesions in food foraging under nonsocial or social contexts, we calculated the total amount of food foraged and eaten in Conditions 1 and 2. A two‐way ANOVA indicated that ACC lesions (*F* (1, 41) = 26.28, *p < *.0001) and social environment (*F* (1, 41) = 6.230, *p < *.05) both affected the amount of food foraged, although no main effect of their interaction (*F* (1, 41) = 0.9785, *p > *.05) was observed (Figure 4a). However, the amount of food eaten was not affected by ACC lesions (*F* (1, 46) = 0.3659, *p > *.05), social environment (*F* (1, 46) = 0.8700, *p > *.05) or their interaction (*F* (1, 46) = 2.716, *p > *.05) (Figure 4b).

### 3.3 ACC lesions had no effect on social interaction or social agonistic behaviors in rats

To exclude an effect of impaired social interactions or social agonistic behaviors induced by ACC lesions on social food foraging behaviors, we examined social interactions and social agonistic behaviors after ACC lesions.

**Figure 5:**
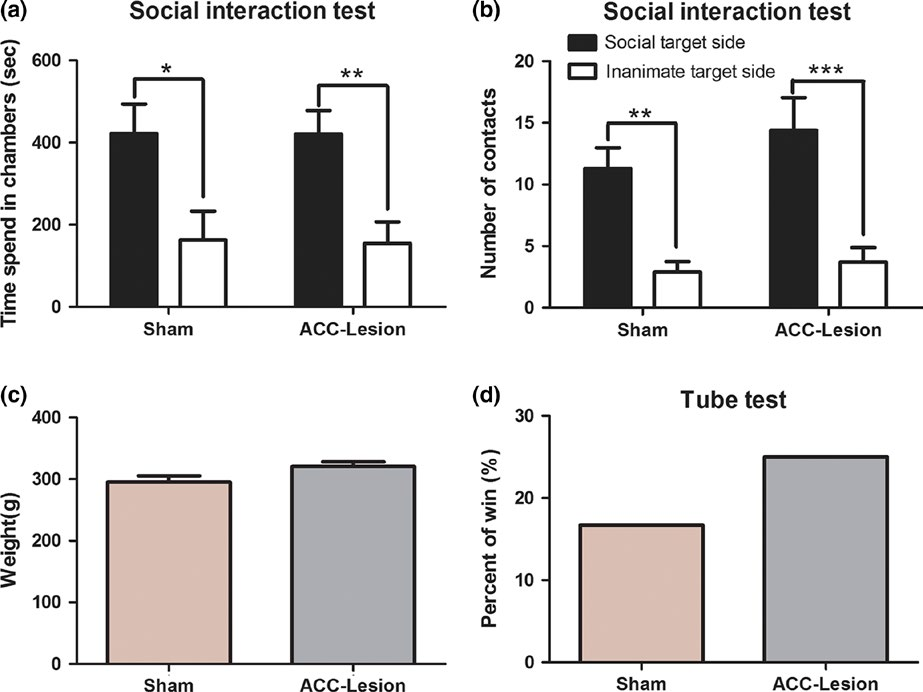
Effects of anterior cingulate cortex (ACC) lesions on the social interaction and social agonistic behaviors in rats. (a) The time spent in chambers. (b) The number of contacts in the social interaction test. The social interaction is indicated by black columns, and inanimate interaction is indicated by white columns. Both the sham group and the ACC‐lesioned group spent more time in and made more contacts with the social target side than the inanimate side. (c) The weight of sham and ACC‐lesioned rats. (d) The percentage of winning in the tube test. There was no significant difference in the weight or the percentage of winning between the sham group and the ACC‐lesioned group. *n* = 12 for each group. ***p < .001, **p < .01 and *p < .05

We used a large three‐chamber glass apparatus to examine the rats' social interaction behavior, as described in our previous report (Cao et al., 2013). Two‐way ANOVA indicated a significant main effect of the side of the box (*F* (1, 30) = 17.71, *p < *.0001) but not of ACC lesions (*F* (1, 30) = 0.005647, *p > *.05) or the interaction term (*F* (1, 30) = 0.002906, *p > *.05) (Figure 5a) on the time the rat spent in the chambers. Likewise, there was a significant main effect of the side of the box (*F* (1, 32) = 27.50, *p < *.0001) but not of ACC lesion (*F* (1, 32) = 1.162, *p > *.05) or the interaction term (*F* (1, 32) = 0.4000, *p > *.05) for the number of the rats' contacts (Figure 5b). Using Bonferroni posttests, we found that there was a significant effect of social interaction in both the ACC‐lesioned group and the sham group on the time spent in and number of contacts with the social target side box (*p < *.05), but there was no significant difference on the time spent in and number of contacts with the social target side box between the two groups (*p > *.05). These results indicated that ACC lesions might have no effect on the social interaction behaviors of rats.

The tube test was used to examine social agonistic behaviors as reported in a previous study (Wang et al., 2011). Before the test, we weighed the rats to ensure their weights were matched, and a *t* test showed there was no significant difference between the sham group (295.0 ± 10.52 g) and the ACC‐lesioned group (320.6 ± 7.47 g) (*t* = 2.042, *p > *.05, *n* = 12 for each group) (Figure 5c). Then, the rats were paired according to their weight (*n* = 12 pairs). The winning percentage of the sham group was 16.7% and for the ACC‐lesioned group it was 25%. A Chi‐square test showed there was no significant difference between the sham group and the ACC‐lesioned group in their winning percentage (Chi‐square=0.8000, *p > *.05) (Figure 5d) in the tube test. These results suggested that ACC lesions might have no effect on the rats' social agonistic behaviors.

## 4 DISCUSSION

Food‐foraging decision‐making behavior of animals in a social setting is a common, critical consideration in nature. In our previous study, we successfully created an experimental model that simulated natural food foraging decision‐making behavior without any artificial intervention or training (Li et al., 2016), yet the brain regions underlying this type of social conflict and benefit decision were unknown. Our present data suggested that the ACC plays a pivotal role in food‐foraging decision‐making in social settings.

Social decision‐making is arguably the most complex cognitive function performed by the brain, and there are two important features of social decision‐making: predicting the outcomes of different actions and considering other members of the social group when choosing an action (Seo & Lee, 2012). In the current study, of which hypothesis was referred the previous research, we used the food foraging decision‐making model in different conditions, which can simulate real‐life situations in nature, e.g., considering the other rats (Conditions 2, 4 and 5) and the value of the food (Conditions 3, 4 and 5). All 5 social situations required rats to make decisions based on the information with which they were confronted. Studies have demonstrated that different decisions were made under social or nonsocial contexts. For example, a recent study indicated that the effect of prior beliefs on stay/leave decision‐making was much less pronounced in a social than in a nonsocial context (Heijne & Sanfey, 2015).

Considerable evidence indicated that the ACC has a central role in action selection in both social and nonsocial contexts. In our present research, we found that ACC lesions‐induced selective alterations in food foraging decision‐making behaviors in rats. As described above, under Condition 2, when faced with a conflict rat in the right side cage, the ACC‐lesioned group foraged less food from the cage with a resident rat than from the empty cage whereas the sham‐lesioned rats preferred to forage food from the cage with a resident rat. These results were similar to the study by Kristin L. Hillman and David K. Bilkey, in which ACC neuronal activity was recorded in freely moving rats as they performed a competitive foraging choice task and ACC‐lesions would lead to 50/50 chance decision‐making (Hillman & Bilkey, 2010). When at least one of the two choice options demanded competitive effort, the majority of ACC neurons exhibited heightened and differential firing between the goal trajectories (Hillman & Bilkey, 2012). Furthermore, in both a nonsocial context (Condition 1) and a social context (Condition 2), the total amount of food foraged was significantly decreased in the ACC‐lesioned group when compared with the sham group. Moreover, in the social context, the total amount of food foraged was less than in the nonsocial context for the ACC‐lesioned animals. We also found that, in both a nonsocial context (Condition 1) and a social context (Condition 2), the amount of food eaten by the ACC‐lesioned group was not significantly different from the amount eaten by the sham group (Figure 4b), which could extend our current and previous foraging findings (Li et al., 2012), suggesting that neither ACC‐lesion nor social context might affect the basic food intake (food intake of the sham rats in Condition 1), even under such circumstances that the amount of foraged food was significantly decreased (Figure 4a). Perhaps the ACC‐lesion animals are more efficient foragers. Recently, one (Hillman & Bilkey, 2012) study suggested that the gyral ACC plays an important role in signaling cost‐benefit information by signaling the value of others' rewards during social interactions (Apps & Ramnani, 2014). These results suggested that the ACC mediates food foraging decision‐making behaviors in social contexts.

Not only social‐based but also value‐based pathways are involved in food‐foraging decision‐making behaviors (Payzan‐LeNestour, Dunne, Bossaerts, & O'Doherty, 2013; Rangel, Camerer, & Montague, 2008; Rilling, King‐Casas, & Sanfey, 2008). Studies have demonstrated that the ACC plays an important role in both social‐based decision‐making (de Araujo et al., 2012; Steinmann et al., 2014) and value‐based decision‐making (Apps & Ramnani, 2014; Massar et al., 2015; Vassena et al., 2014). In Condition 2, the sham rats preferred the reward paired with a stranger rat more than they preferred the same reward without any rats, as same as they do in nonforaging contexts (Figure 5), indicating normal rats found the company of other rats to be rewarding or at least highly predictive of reward. Condition 2 was akin to the barrier task used by Walton, in which a large reward is located behind a large barrier in one arm of a T‐maze, and a small reward is located behind a small barrier in the other arm (Walton et al., 2002). In contrast, ACC lesions inverted the decision‐making process, which seemed to impair the reward of the social‐based pathways. In Condition 3, we used sweet food as reward of value‐based pathways, and rats with ACC lesions selected the value reward as the optimal choice, as did the sham‐lesioned animals, suggesting that ACC lesions had no effect on appetitive behavior.

When high‐value food was presented in the presence of a social conflict under Conditions 4, which was in agreement with our previous study (Li et al., 2016), the sham group foraged a similar percentage of standard food pellets from the rat‐residing cage and sweet food from the no‐conflict cage. The alternative value‐based reward could induce the possibility that the sham rats to select the reward of social‐based choice, or in other words they forwent high‐value rewards to forage for lesser rewards that are nearby other stranger rats, with the possible reasons that this was a high reward and high effort choice as a good default strategy in natural food foraging approach, suggesting the social‐based pathways was equal to the value‐based pathways in normal rats. In Condition 5, sham‐lesioned animals preferred foraging for high‐value food from the cage with a resident rat. However, when faced with high‐value food in the conflict cage and standard food in the empty cage, the ACC‐lesioned group foraged more standard food than sweet food, indicating the effect of ACC lesions was not only impairing the social encountered reward, but also negative feedback from competitive effort in social foraging contexts, which was consistent with the study by Hillman & Bilkey (2010). All of these indicate that ACC lesions cause the group‐housed rats to switch the choice from the high value reward with high social competitive effort to the low value reward with low social competitive effort in food foraging decision‐making strategies.

To eliminate the possibility that the effects of ACC lesions on food‐foraging decision‐making were due to their effects on social interactions and social agonistic behaviors, social interaction and tube tests were performed after ACC lesions in this study. Both the sham rats and the ACC‐lesioned rats were happy to socialize with, or to confront other same sex stranger rats in nonforaging contexts. Social interactions and social agonistic behaviors were not affected by ACC lesions, which was inconsistent with the study by Peter H. Rudebeck, whose results showed ACC lesions affected the utilization of social information, diminished social behavior, and reduced social stimulating memory, whereas normal rats habituated to socialize with other rats (Rudebeck et al., 2007), as well as another recent study reported that activity in the ACC was positively related to response to aggressive behavior (Beyer, Munte, Gottlich, & Kramer, 2015). These inconsistent results were probably due to the different procedures used to assess social interaction behavior as well as variability in lesion locations. Our previous study also showed that ACC lesions had no effect on the rat's anxiety and depression‐like behaviors (Li et al., 2012), which further indicated that the change in food foraging decision‐making produced by ACC lesions in our study were not due to the dysfunction of social interaction and social agonistic behaviors.

This study provides strong support that ACC lesions inverted the decision‐making process in social‐based and but not in value‐based food foraging, and these alterations are not due to impaired social agonistic or social interaction behaviors, without effect on basic food intake. All these results indicate that the ACC is a key brain region underlying neural processing of social food foraging decision‐making, specifically tending to compete for foraging high predictive reward food.

## CONFLICT OF INTEREST

The authors declare no conflict of interest.
